# Supplementary material for: Leveraging transcript quantification for fast computation of alternative splicing profiles
Source: RNA. 2015 Sep;21(9):1521–31. doi: 10.1261/rna.051557.115 (PMC4536314; doi:10.1261/rna.051557.115)
Supplement: Supplemental Material [file supp_21_9_1521__index.html]

Leveraging transcript quantification for fast computation of alternative splicing profiles — Leveraging transcript quantification for fast computation of alternative splicing profiles — Supplemental Material 

# Leveraging transcript quantification for fast computation of alternative splicing profiles

## Supplemental Material

**Files in this Data Supplement:**

- Supp Material.pdf
- Supp Data 1.xls
- Supp Data 5.xls
- Supp Data 4.xls
- Supp Data 2.xls
- Supp Data 3.xls
