# Supplementary material for: Leveraging transcript quantification for fast computation of alternative splicing profiles
Source: RNA. 2015 Sep;21(9):1521–31. doi: 10.1261/rna.051557.115 (PMC4536314; doi:10.1261/rna.051557.115)
Supplement: Supplemental Material [file supp_051557.115_SuppMaterial_supp_v18.pdf]

Supplementary Material

Leveraging transcript quantification for fast computation of alternative splicing profiles

Gael P. Alamancos[1,*], Amadís Pagès[1,4,*], Juan L. Trincado[1], Nicolás Bellora[2], Eduardo Eyras[1,3,5]

Universitat Pompeu Fabra, E08003, Barcelona, Spain

[2]INIBIOMA, CONICET-UNComahue, Bariloche, Río Negro, Argentina.

[3]Catalan Institution for Research and Advanced Studies, E08010 Barcelona, Spain

[4]Centre for Genomic Regulation, E08003, Barcelona, Spain

[5]corresponding author: eduardo.eyras@upf.edu

[*]These authors contributed equally

Accuracy analysis with simulated data separated by event type

Event Type	*Events Num*	Sailfish+SUPPA		RSEM+SUPPA		MATS		MISO	
		Pearson	*Spearman*	*Pearson*	*Spearman*	*Pearson*	*Spearman*	*Pearson*	*Spearman*
SE	94	0,9679	0,9560	0,9848	0,9765	0,8048	0,8012	0,8502	0,7964
A3	23	0,9682	0,9294	0,9877	0,9644	0,8387	0,8113	0,8637	0,8187
A5	15	0,9938	0,9336	0,9936	0,9387	0,9906	0,9184	0,9351	0,9005
MX	4	0,9998	1,0000	0,9996	1,0000	0,5343	0,1054	0,6026	0,8333
RI	8	0,9981	0,8503	0,9979	0,9048	0,9624	0,8810	0,9944	0,8193

Supplementary Table 1. Correlation values (Spearman and Pearson R) between the estimated and ground-truth Ψ values using simulated data. The comparison involves 144 events (Supplementary Data 1) separated by type: skipping exon (SE), alternative 3' (A3) and 5' (A5) splice-site, mutually exclusive (MX) and retained intron (RI).

Comparison of distributions of the absolute differences between predicted and reference inclusion values

Comparison	*Synthetic Data*	*RT-PCR*	
		ESRP1	*EV*
SUPPA + Sailfish vs MATS	8,89E-012	0,9226	0,6498
SUPPA + Sailfish vs MISO	1,86E-013	0,4296	0,3024
SUPPA + RSEM vs MATS	2,72E-016	0,9812	0,9247
SUPPA + RSEM vs MISO	2,20E-016	0,3225	0,1273

Supplementary Table 2. Statistical comparison between the distributions of absolute differences between predicted and reference Ψ values. For the synthetic data we use the ground truth generated as described in the text. For the experimental datasets we use the RT-PCR values from (Shen et al. 2012). The P-values correspond to a P-value unpaired Wilcoxon Rank-Sum Test (Mann-Whitney) comparing the datasets rrelation values (Spearman and Pearson R) between the estimated and ground-truth Ψ values using simulated

RNA sequencing of cytosolic fractions from MCF7 and MCF10 cells

MCF7 cells were cultured in Dulbecco's modified Eagle's medium (DMEM) supplemented with 10% fetal bovine serum (FBS) and penicillin/streptomycin. MCF10 cells were cultured in DMEM/F12 supplemented with 15 mM hepes buffer, 10% FBS, 10 mg/ml insulin, 25 ng/ml EGF, 100 ng/ml choleratoxin and 0.5 mg/ml hydrocortisone. Cell fractionation was performed with PARIS Kit from Ambion following their instructions. Two biological replicates from each sample were sequenced with Illumina HiSeq-2000 using a standard stranded protocol. Sequencing reads are available at SRA (SRP045592). We obtained an approximate amount of 42M and 46M paired-end reads for the two cytosolic MCF7 replicates, and 45M and 56M paired-end reads for the two cytosolic MCF10 replicates.

	**RefSeq**	**Ensembl**
A3SS	3270	13226
A5SS	2943	11808
SE	8764	31974
RI	794	4797
MXE	943	4772
Total	16714	66577

Supplementary Table 3. Number of alternative splicing events obtained by running the *generateEvents* operation of SUPPA on the RefSeq and Ensembl annotations. The table provides the totals and the number of each type of event: alternative 3' (A3) and 5' (A5) splice-sites, exon skipping (SE), intron retention (RI) and mutually exclusive events (MX) as described in Figure 1. Alternative first and last exons were not included in the analysis but can be also computed with SUPPA (https://bitbucket.org/regulatorygenomicsupf/suppa/).

**Gene Expression Parameters**	REF_FILE_NAME	/pathTo/refseq_filtered_annotation.gtf
	LOAD_CODING	True
	LOAD_NONCODING	True
	NB_MOLECULES	5.000.000
	EXPRESSION_K	-0.6
	EXPRESSION_X0	9.500
	EXPRESSION_X1	9.500^2
	TSS_MEAN	25
	POLYA_SCALE	NaN
	POLYA_SHAPE	NaN
**Reverse transcription Parameters**	RTRANSCRIPTION	True
	RT_PRIMER	RH
	RT_MIN	500
	RT_MAX	5.500
**Fragmentation Parameters**	FRAGMENTATION	true
	FRAG_METHOD	UR
	FRAG_SUBSTRATE	RNA
	FRAG_UR_D0	1
	FRAG_UR_DELTA	NaN
	FRAG_UR_ETA	NaN
**Final Library Preparation Parameters**	FILTERING	True
	PCR_PROBABILITY	0.05
	GC_MEAN	NaN
	GC_SD	NaN
**Sequencing Parameters**	READ_NUMBER	90.000.000
	READ_LENGTH	50
	PAIRED_END	True
	GEN_DIR	/pathTo/hg19_fasta/
	FASTA	True
	ERR_FILE	76
	UNIQUE_IDS	False

Supplementary Table 4 Simulated human RNA-Seq data was generated with FluxSimulator using the command: "/pathTo/flux-simulator-1.2.1/bin/flux-simulator -x -l -s -p /pathTo/parameters_file.par" with the parameters listed in the Table. This resulted in a dataset of 45 million 2x76bp paired-end reads, which were split into two different FASTQ files, one containing the paired-end reads mapping in the sense strand of the transcripts and the other one with the paired-end reads in the antisense strand.

	Synthetic data	MDA-MB-231 (ESRP1+EV)	MCF7 (rep1+rep2)	MCF10 (rep1+rep2)
A3	307	1651	1348	1234
A5	259	1436	1251	1141
SE	1401	19232	10502	7668
RI	74	504	418	383
MX	274	3193	1221	811
Total	2315	26016	14740	11237

Supplementary Table 5. Number of alternative splicing events for which MATS reported a Ψ_{MATS} value using the RefSeq annotation, for each of the experiments used. The table provides the totals counts and the number of each type of event: alternative 3' (A3) and 5' (A5) splice-sites, exon skipping (SE), intron retention (RI) and mutually exclusive events (MX) as described in Figure 1. Since MATS requires 2 experiments, the values in each of the columns correspond to using as input twice the same file for the synthetic data, the ESRP1 and EV samples for the MDA-MB-231 data, the two replicates for the cytosolic MCF7 samples, and the two replicates for the cytosolic MCF10 samples.

	Synthetic data	ESRP1	EV	MCF7 (rep 1)	MCF7 (rep 2)	MCF10 (rep 2)	MCF10 (rep 2)
A3SS	1957	2185	2103	1916	1850	1753	1905
A5SS	1791	2154	2061	1945	1929	1800	1933
SE	5418	5795	5575	5259	5072	4829	5175
RI	475	574	554	545	533	505	534
MXE	735	647	622	594	585	567	593
Total	10376	11355	10915	10259	9969	9454	10140

Supplementary Table 6. Number of alternative splicing events for which MISO reported a Ψ_{MISO} value using the RefSeq annotation, for each of the experiments used. The table provides the total counts and the number of each type of event: alternative 3' (A3) and 5' (A5) splice-sites, exon skipping (SE), intron retention (RI) and mutually exclusive events (MX) as described in Figure 1. Since MISO can accept only 1 single experiment, we give the values for each sample separately.

	Synthetic data	ESRP1	EV	MCF7 (rep 1)	MCF7 (rep 2)	MCF10 (rep 1)	MCF10 (rep 2)
A3SS	2181	2232	2253	2232	2238	2217	2229
A5SS	1930	2181	2197	2213	2196	2182	2184
SE	5880	5768	5820	5905	5875	5803	5834
MX	565	556	565	577	586	528	552
RI	490	575	579	576	572	558	558
Total	11046	11312	11414	11503	11467	11288	11357

Supplementary Table 7. Number of alternative splicing events for which SUPPA reported a Ψ value using the RefSeq annotation with Sailfish, for each of the experiments used quantification and restricting to genes with TPM >= 1, where gene TPM is calculated as the total TPM of the transcripts in the gene. The table provides the total counts and the number of each type of event: alternative 3' (A3) and 5' (A5) splice-sites, exon skipping (SE), intron retention (RI) and mutually exclusive events (MX) as described in Figure 1A. For MCF7 and MCF10 samples, events for both cytosolic replicates are reported.

	Synthetic data	ESRP1	EV	MCF7 (rep 1)	MCF7 (rep 2)	MCF10 (rep 1)	MCF10 (rep 2)
A3SS	2100	2157	2205	2182	2193	2148	2143
A5SS	1829	2182	2154	2178	2176	2123	2119
SE	5617	5493	5654	5771	5785	5659	5634
MX	525	555	576	567	584	529	562
RI	466	559	563	563	562	542	536
Total	10537	10946	11152	11261	11300	11001	10994

Supplementary Table 8. Number of alternative splicing events for which SUPPA reported a Ψ value using the RefSeq annotation with RSEM quantification, for each of the experiments used and restricting to genes with TPM >= 1, where gene TPM is calculated as the total TPM of the transcripts in the gene. The table provides the total counts and the number of each type of event: alternative 3' (A3) and 5' (A5) splice-sites, exon skipping (SE), intron retention (RI) and mutually exclusive events (MX) as described in Figure 1. For MCF7 and MCF10 samples, events for both cytosolic replicates are reported.

Analysis	Step	Command
**(A)** Generation of $Y_{Sailfish}$ values	**(A1)** Generation of Sailfish index	/pathTo/sailfish-0.6.3/bin/sailfish index -t /pathTo/refseq_filtered_annotation.fasta -k 31 -o /pathTo/sailfishIndexDir/
	**(A2)** Transcript quantification with Sailfish	/pathTo/sailfish-0.6.3/bin/sailfish quant -i /pathTo/sailfishIndexDir/ -l "T=PE:O=>>:S=U" -1 /pathTo/mcf7r1_reads_1.fastq -2 pathTo/mcf7r1_reads_2.fastq -o /pathTo/SailfishQuantificationOutput/
	**(A3)** Generation of alternative splicing events with SUPPA	/pathTo/python-2.7/python /pathTo/SUPPA/suppa.py generateEvents -i /pathTo/refseq_filtered_annotation.gtf -o /pathTo/SuppaEventsOutput/event -e SE SS MX RI
	**(A4)** Quantification of $Y_{Sailfish}$ values with SUPPA	**Command 1**<br>/pathTo/python-2.7/python /pathTo/SUPPA/multipleFieldSelection.py -i /pathTo/SailfishQuantificationOutput/quant.sf -k 1-f 3 -o /pathTo/SailfishQuantificationOutput/mcf7r1_tpm.tab<br><br>**Command 2**<br>/pathTo/python-2.7/python /pathTo/SUPPA/suppa.py psiPerEvent -i /pathTo/SuppaEventsOutput/event_SE.ioe -e /pathTo/SailfishQuantificationOutput/mcf7r1_tpm.tab -o /pathTo/Suppa-SailfishPsiFolder/mcf7r1_psi_se.tab

Supplementary Table 9. Commands to generate the $Y_{Sailfish}$ values, i.e. the SUPPA Y values based on the Sailfish transcript quantification. We provide the specific example of using RefSeq annotation and the MCF7 samples. Other samples and annotations can be run analogously. For the MCF7 samples, steps A2 and A3 would be repeated for each replicate. Command 2 from step A4 would be repeated for the files corresponding to each type of event: SE, A3, A5, MX and RI.

Analysis	Step	Command
**(B)** Generation of Y_{MATS} values	**(B1)** Generation of Y_{MATS} values from raw read data with MATS	/pathTo/python-2.7/bin/python /pathTo/MATS.3.0.1.beta/RNASeq-MATS.py -s1 /pathTo/mcf7r1_reads_1.fastq:/pathTo/mcf7r1_reads_2.fastq -s2 /pathTo/mcf7r2_reads_1.fastq:/pathTo/mcf7r2_reads_2.fastq -gtf /pathTo/refseq_filtered_annotation .gtf -o /pathTo/MatsOutput/ -bi /pathTo/bowtieIndex/hg19 -t paired -len 50 -a 8 -c 0.05 -expressionChange 10.0 -analysis U

Supplementary Table 10. Command to generate the Y values with MATS. We provide the specific example of using RefSeq annotation and the MCF7 samples. Other samples and annotations can be run analogously.

Analysis	Step	Command
**(C)** Generation of Y_{MISO} values	**(C1)** Format conversion	Conversion of alternative splicing events generated in step **(A3)** to MISO's GFF-based alternative events format
	**(C2)** Preparation of alternative isoforms annotation	/pathTo/MISO/index_gff --index /pathTo/misoEvents/SE.gff3 /pathTo/misoIndexedEvents/SE<br><br>[Note] This command is repeated for files corresponding to A3, A5, MX and RI events
	**(C3)** Conversion of alignment files in SAM format obtained in step **(B1)** to sorted BAM files	**Command 1**<br>/pathTo/samtools-0.1.18/samtools view -S -h -b -o /pathTo/bamFiles/mcf7r1.bam /pathTo/MatsOutput/mcf7r1.sam<br><br>**Command 2**<br>/pathTo/samtools-0.1.18/samtools sort /pathTo/bamFiles/mcf7r1.bam /pathTo/bamFiles/mcf7r1.sorted.bam
	**(C4)** Generation of Y_{MISO} values from BAM files with MISO	**Command 1**<br><br>/pathTo/MISO/miso --run /pathTo/misoIndexedEvents/SE /pathTo/bamFiles/mcf7r1.sorted.bam --output-dir /pathTo/MisoOutput/ --read-len 50<br><br>**Command 2**<br><br>/pathTo/MISO/summarize_miso --summarize-sample /pathTo/MisoOutput/SE.out /pathTo/MisoSummarizedOutput/SE.summary

Supplementary Table 11. Commands to generate the Y values from MISO. We provide the specific example of using RefSeq annotation and the MCF7 samples. Other samples and annotations can be run analogously. For the MCF7 samples, steps C3 and C4 would be repeated for each replicate. Both commands from step C4 would be repeated for the files corresponding to each type of event: SE, A3, A5, MX and RI.

**Analysis**	**Step**	**Command**
**(D)** Generation of Y_{RSEM} values	**(D1)** Preparation of RSEM reference file	/pathTo/rsem-1.2.19/rsem-prepare-reference –bowtie2 /pathTo/refseq_filtered_annotation.fasta /pathTo/RsemReference/refseq
	**(D2)** Transcript quantification with RSEM	/pathTo/rsem-1.2.19/rsem-calculate-expression --bowtie2 --paired-end /pathTo/mcf7r1_reads_1.fastq /pathTo/mcf7r1_reads_2.fastq /pathTo/RsemReference/refseq /pathTo/RsemQuantificationOutput/
	**(D3)** Quantification of Y_{RSEM} values with SUPPA against events obtained in (A3)	**Command 1**<br>/pathTo/python-2.7/python /pathTo/SUPPA/multipleFieldSelection.py -i /pathTo/RsemQuantificationOutput/mcf7r1 .isoforms.results -k 1-f 6 -o /pathTo/RsemQuantificationOutput/mcf7r1_tpm.tab<br><br>**Command 2**<br>/pathTo/python-2.7/python /pathTo/SUPPA/suppa.py psiPerEvent -i /pathTo/SuppaEventsOutput/event_SE.ioe -e /pathTo/RsemQuantificationOutput/mcf7r1_tpm.tab -o /pathTo/Suppa-RsemPsiFolder/mcf7r1_psi_se.tab

Supplementary Table 12. Commands to generate the Y_{RSEM}, i.e. the SUPPA Y values based on the RSEM transcript quantification. We provide the specific examples of using RefSeq annotation and the MCF7 samples. Other samples and annotations can be run analogously. For the MCF7 samples, steps D2 and D3 would be repeated for replicate 2. Command 2 from step D3 would be repeated for the files corresponding to each type of event: SE, A3, A5, MX and RI.

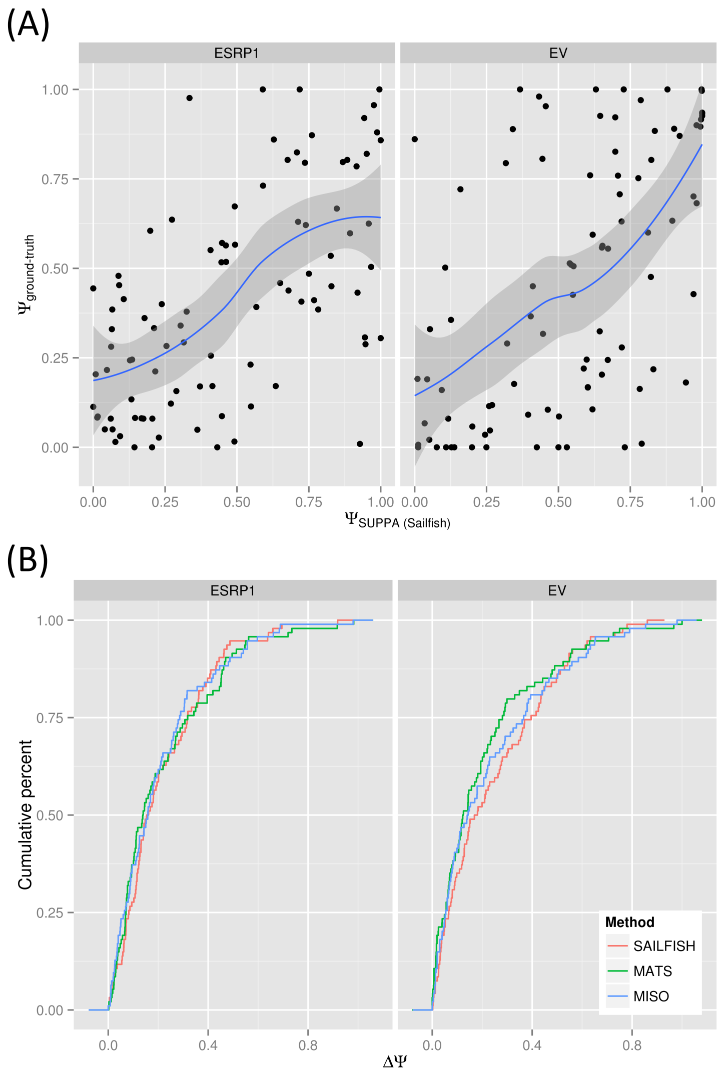

Supplementary Figure 1. Benchmarking experimentally validated events using Ensembl annotation. (A) Correlation of the experimental Ψ values with those estimated with Sailfish+SUPPA in MDA-MB-231 cells with (ESRP1, left panel) and without (EV, right panel) ESRP1 overexpression. Experimental Ψ values were obtained by RT-PCR (Shen et al. 2012) and estimated PSIs were obtained from RNA-Seq data in the same samples (Shen et al. 2012). The blue curve and gray boundaries are the fitted curves with the LOESS regression method. **(B)** Cumulative distribution of the absolute difference between the same experimental Ψ values and the ones estimated with Sailfish+SUPPA (SAILFISH), RSEM+SUPPA (RSEM), MISO and MATS from RNA-Seq data under the same conditions. The lines describe the proportion of all events (Cumulative percent, y-axis) that are calculated at a given maximum absolute difference (**ΔΨ**, x-axis) from the experimental. Third panel shows the comparison between the ground-truth Ψ values in a simulated dataset and the estimated Ψ values.

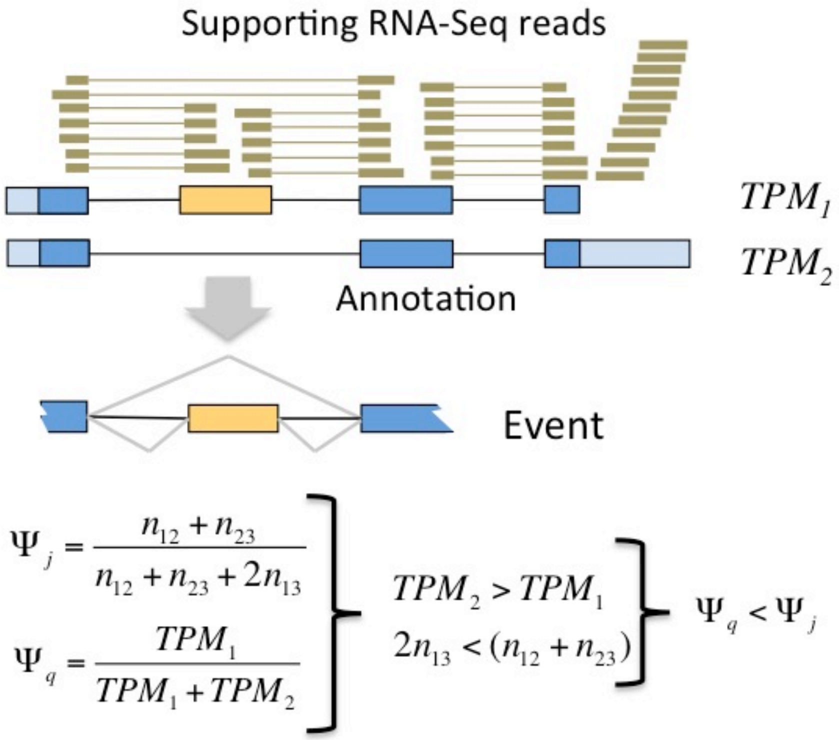

Supplementary Figure 2. In the figure we illustrate a gene with two transcript isoforms, where only one of them has a 3' UTR fully annotated. Quantification of the transcripts may give a higher TPM to transcript 2 ($TPM_2 > TPM_1$) due to a large amount of reads at the 3'UTR. However, junction reads may give the opposite result. In the figure, according to the junction reads, transcript 1 appears to have greater support. As a consequence, the PSI calculation according to junction reads will give a high inclusion to the alternative exon, i.e. $\Psi_j \sim 1$. On the other hand, since the transcript that skips the exon gets larger quantification, i.e. $TPM_2 > TPM_1$, PSI calculation based on transcript expression would give the opposite behaviour, i.e. $\Psi_q << 1$.

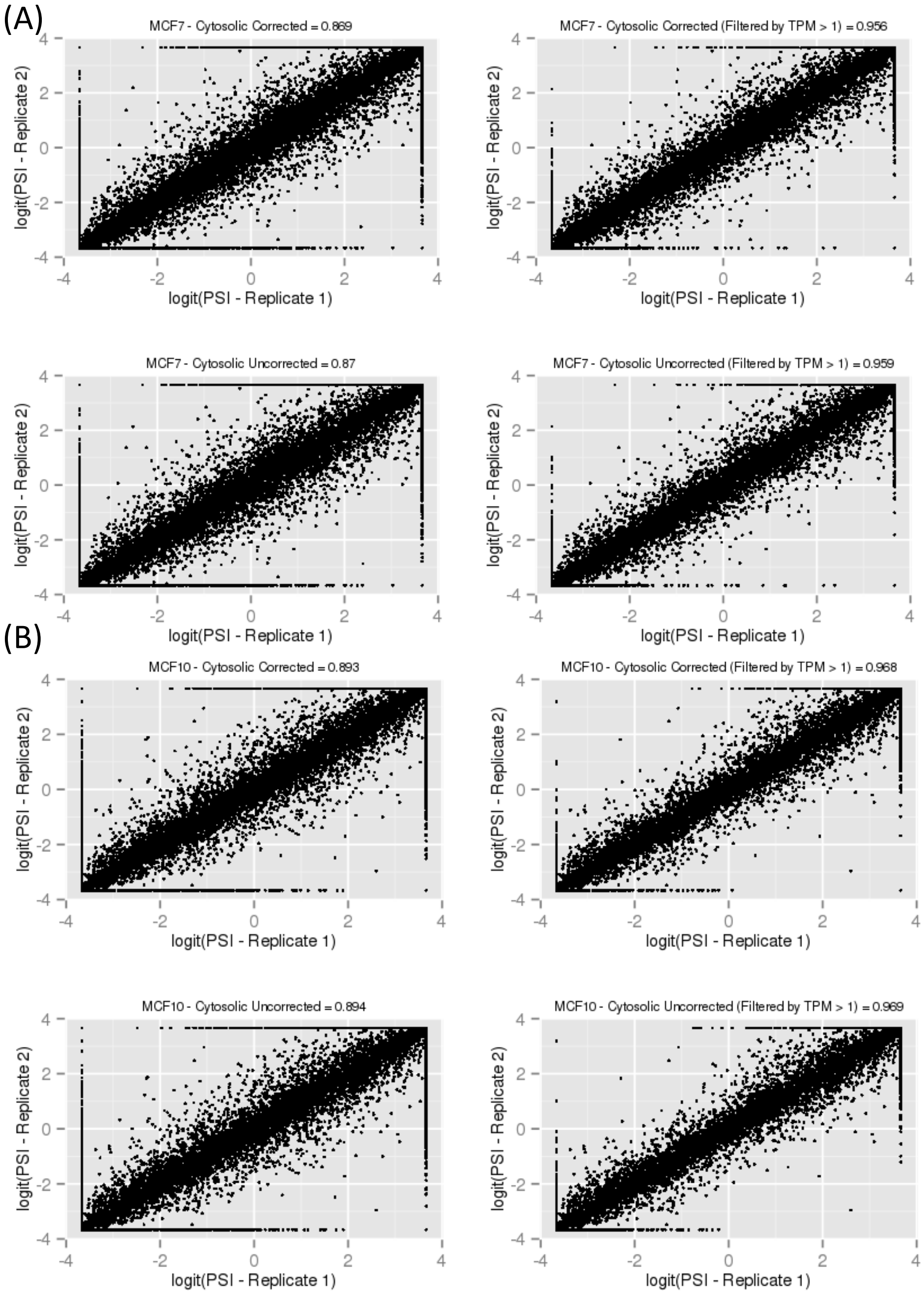

Supplementary Figure 3. Correlation between PSI values (logit-transformed) for the replicates of the cytosolic MCF7 **(A)** and MCF10 **(B)** cytosolic RNA-Seq samples. Upper panels use corrected TPM from Sailfish, whereas the lower panels use the uncorrected values. Right panels show the correlations for events in genes with TPM>1, left panels show the correlations without filtering

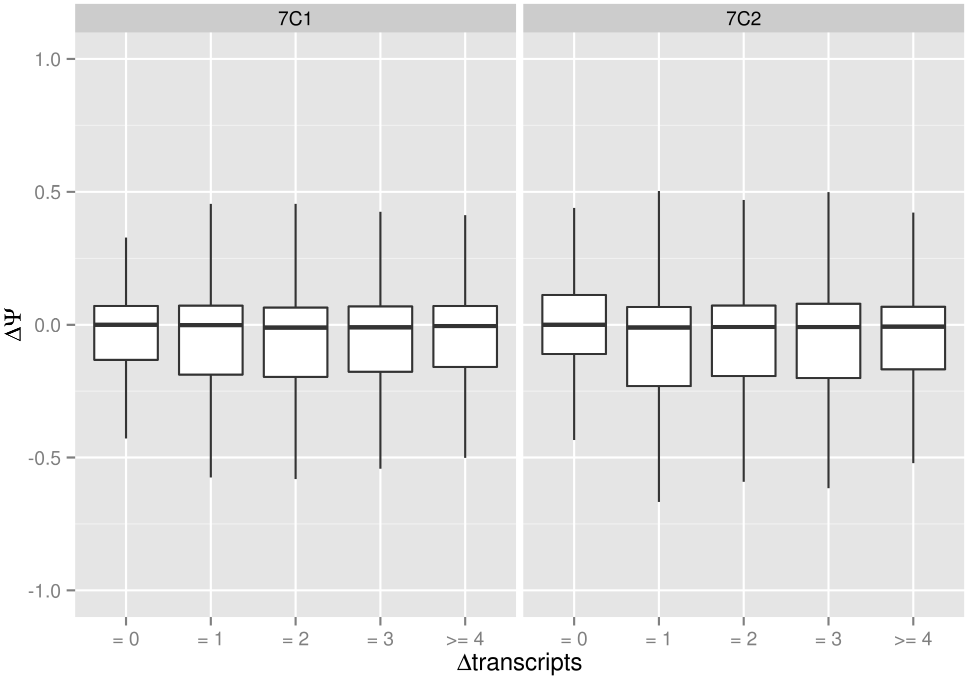

Supplementary Figure 4. Annotation dependencies. Boxplots of the difference Ψ value between SUPPA estimates using Sailfish quantification on Ensembl and RefSeq annotation (y axis), as a function of difference in the number of transcripts annotated per gene (x axis), for the genes in which the events are contained**.**
